# Chatbot Usability Scale in Chinese Users: Cross-Cultural Adaptation and Validation Study

**DOI:** 10.2196/84971

**Published:** 2026-04-06

**Authors:** Haoming Ma, Runyuan Pei, Sijia Li, Aoqi Wang, Xingyi Tang, Meihua Piao

**Affiliations:** 1 School of Nursing, Peking Union Medical College, Chinese Academy of Medical Sciences, 33 Badachu Road, Beijing, 100144, China, 86 13522112889

**Keywords:** chatbot, usability, satisfaction, user experience, cross-cultural adaptation

## Abstract

**Background:**

Chatbots are increasingly deployed across various domains; however, systematic evaluation of their usability remains limited, particularly in non-Western contexts. The 11-item Chatbot Usability Scale (BUS-11), a multidimensional instrument grounded in human-computer interaction theory, has demonstrated strong psychometric properties in prior studies; however, a validated Chinese version does not exist, despite China being one of the largest chatbot markets.

**Objective:**

This study aimed to translate, culturally adapt, and validate the BUS-11 for Chinese users.

**Methods:**

Following established cross-cultural adaptation procedures, the scale was forward- and back-translated, reviewed by an expert committee, and pilot-tested for clarity and feasibility. A main validation study was then conducted with 214 participants, who completed 438 evaluations of the chatbots across 10 widely used systems.

**Results:**

Psychometric analyses demonstrated excellent scale-level content validity index (ie, 0.92), strong internal consistency (Cronbach α=0.92), and a clear 3-factor structure (Accessibility, Interaction Process Quality, and Information Quality), explaining 56.1% of the variance. Meanwhile, privacy or security and response time were retained as single-item indicators. The Chinese BUS-11 was concise, user-friendly, and psychometrically robust.

**Conclusions:**

This work fills a critical gap by providing the first validated instrument for assessing chatbot usability in Chinese contexts, enabling reliable cross-cultural comparisons and supporting both research and practical design evaluation in human-computer interaction.

## Introduction

Chatbots (conversational agents) are artificial intelligence systems designed to interact with users through natural language dialog [1]. They have become increasingly ubiquitous across the globe in domains ranging from online retail and customer service to public administration and health care [2-4]. China represents a particularly active context for chatbot adoption, with large-scale use in commercial platforms, government service portals, and hospital-based patient guidance systems [5-7]. As chatbots become embedded in everyday services, their usability becomes increasingly consequential because it shapes user satisfaction, acceptance, and sustained engagement [8].

In human-computer interaction (HCI), usability refers to how easy, efficient, and satisfying a system is for users to achieve their goals [9]. For chatbots, usability extends beyond general ease of use to include interaction qualities that are specific to conversation—such as turn-taking, dialog coherence, response appropriateness, and perceived trustworthiness [10,11]. However, commonly used usability instruments (eg, the System Usability Scale) were developed for graphical user interfaces and provide limited coverage of conversational properties central to chatbot interaction [12]. To better reflect the chatbot context, the Chatbot Usability Questionnaire (CUQ) adapts traditional usability constructs to conversational agents, emphasizing ease of use, navigability, clarity of onboarding, and basic error handling [13]. Yet, the CUQ remains anchored in general usability notions and may insufficiently capture AI-specific determinants of sustained chatbot use, including information adequacy, contextual understanding, responsiveness, and privacy- and trust-related concerns.

To address these gaps, Borsci et al [10,14] proposed the 11-item Chatbot Usability Scale (BUS-11), a theory-driven, chatbot-native instrument developed from HCI principles and validated through multistage psychometric testing across different chatbot systems and contexts. The BUS-11 operationalizes chatbot usability as a multidimensional construct encompassing Accessibility, Interaction Process Quality, Information Quality, Privacy and Security, and Response Time, thereby facilitating comprehensive evaluation and cross-system comparability. Despite its growing international adoption, a validated Chinese version of the BUS-11 is currently lacking, which limits the rigorous measurement of usability in one of the world’s largest chatbot markets.

Cross-cultural adaptation of usability instruments requires ensuring semantic, conceptual, and cultural equivalence to preserve measurement validity across contexts [15]. Building on established adaptation guidelines and prior localization efforts in Chinese HCI research [16], this study aims to translate and culturally adapt the BUS-11 into Chinese and to evaluate its psychometric properties. Specifically, we (1) conducted a rigorous translation and cultural adaptation of the BUS-11, (2) examined reliability, validity, and factor structure using data from 214 participants and 438 chatbot evaluations, and (3) provided initial evidence on Chinese users’ perceptions of chatbot usability to facilitate future cross-cultural comparisons in chatbot user experience (UX) research [17].

## Methods

### Scale Translation and Cultural Adaptation

#### Overview

Following authorization from the original scale’s corresponding author, the translation of the BUS-11 into Chinese was conducted using the Brislin translation model as improved by Jones et al [18]. Consistent with established guidance for cross-cultural instrument adaptation (eg, forward-back translation with expert reconciliation), we documented all translation decisions to ensure semantic and conceptual equivalence. The specific steps are as follows: (1) forward translation: the researcher and a bilingual professional proficient in both Chinese and English independently translated the original BUS-11 into Chinese. Discrepancies and alternative phrasings were recorded in a reconciliation log and resolved through consensus, resulting in an initial Chinese draft; (2) back translation: another bilingual translator, whose native language was Chinese, translated the initial Chinese draft back into English, producing a back-translated English version; (3) consistency check: a monolingual English-speaking expert familiar with the BUS-11 reviewed and compared the original scale with the back-translated version. Any residual inconsistencies (eg, colloquialisms or culture-specific expressions) were discussed in joint meetings with all translators until consensus was achieved, ensuring conceptual and semantic consistency; (4) final translation: a final Chinese translation was prepared collaboratively by all translators, incorporating adjustments from the consistency review, thus establishing semantic equivalence with the original BUS-11. Minor wording refinements were made for clarity and idiomatic naturalness (no item content was substantively altered). At the end of the cultural adaptation process, the final Chinese version of the BUS-11 and the original English items are presented side by side in Table 1 to facilitate direct comparison of item wording and to enhance the transparency of the adaptation procedure.

**Table 1.** Original English and Chinese versions of the 11-item Chatbot Usability Scale (BUS-11).

Dimension in English (Chinese)	Item in English (Chinese)
Access the chatbot’s functions (一, 访问聊天机器人的功能)	The chatbot function was easily detectable (容易找到此聊天机器人的各种功能 [例：修改聊天机器人使用的语言或修改头像]) It was easy to find the chatbot (找到此聊天机器人的入口是一件简单的事)
Quality of chatbot functions (二, 聊天机器人功能的质量)	Communicating with the chatbot was clear (与此聊天机器人的沟通过程是顺畅的) The chatbot was able to keep track of context (此聊天机器人能够保持上下文的连贯性) The chatbot’s responses were easy to understand (此聊天机器人的回复易于理解)
Provide quality of conversation and information (三, 提供对话和信息的质量)	I find that the chatbot understands what I want and helps me achieve my goal (我感觉此聊天机器人理解我的目的，并协助我达成目的) I believe the chatbot informs me of any possible privacy issues (此聊天机器人提供的信息量是合适的，不多不少) The chatbot only gives me the information I need (此聊天机器人只提供了我需要的信息) I feel like the chatbot’s responses were accurate (我觉得此聊天机器人的回复内容正确无误)
Privacy and security (四, 隐私和安全)	I believe the chatbot informs me of any possible privacy issues (我相信此聊天机器人会告知我任何潜在的隐私问题)
Response time (五, 回复时间)	My waiting time for a response from the chatbot was short (我等待聊天机器人回复的时间是短的)

#### Cultural Adaptation

In June 2024, we convened an expert committee (n=6) to evaluate the cultural appropriateness of the Chinese translation. The expert committee comprised 6 specialists drawn from the broader panel reported in Table 2, all of whom had doctoral-level training or senior professional experience in HCI, usability or UX evaluation, health informatics, or scale development. Eligibility criteria included: (1) formal training in a relevant discipline, (2) demonstrated experience in chatbot-related research or usability evaluation, and (3) prior involvement in scale development, instrument adaptation, or related methodological work. Each expert independently compared the Chinese items with the original BUS-11 and assessed semantic and conceptual equivalence, idiomatic appropriateness, and readability. Disagreements were resolved through iterative discussion until consensus was reached. Revisions focused on wording-level refinements to improve clarity and align the tone with standard Chinese conversational norms, without altering the intended coverage of the construct.

**Table 2.** Background information of the experts.

Expert	Sex	Age (years)	Education	Experience (years)	Domains	Title
1	Male	37	Doctorate	5	Human-Computer Interaction	Researcher
2	Male	38	Doctorate	3	Human-Computer Interaction	Associate Researcher
3	Male	31	Doctorate	1	Health Informatics	Associate Researcher
4	Male	33	Doctorate	6	Health Informatics	Associate Researcher
5	Male	50	Doctorate	27	Computer Science	Professor
6	Male	36	Doctorate	12	Usability/UX^a^ Evaluation	Associate Researcher
7	Female	42	Doctorate	15	Health Informatics	Associate Researcher
8	Male	34	Doctorate	8	Chatbot Systems Engineering	Senior Engineer

a UX: user experience.

### Pilot Study

#### Overview

To ensure participants evaluated chatbots after a realistic, standardized interaction, we designed representative use-case tasks for 10 commonly used chatbot systems in China (refer to Table 3 for details). For example, participants were instructed to interact with Taobao’s intelligent customer service, simulating a typical query related to membership benefits: “Your friend recommends you purchase Taobao’s 88vip. Please use Taobao’s chatbot ‘Smart Customer Service Xiao Mi’ to help you understand the specific benefits of 88vip. Log in as instructed and converse with this chatbot.” Refer to Multimedia Appendix 1 for details. Immediately after completing the assigned task, participants filled out the Chinese BUS-11 about that chatbot. The same task-rating flow was later applied in the main study.

**Table 3.** Overview of the chatbots evaluated in the study. General-purpose large language model (LLM)-based chatbots (eg, Doubao, Wenxin Yiyan, and Kimi) were evaluated using a single domain-specific task to ensure consistency with the task-based evaluation applied to all systems.

Chatbot (developers)	Domain	Platform	Primary function	Example task
AliMe (Taobao; Alibaba)	E-commerce	App or web	Customer service	Understanding Taobao 88VIP benefits
Zhihu Zhida (Zhihu Inc)	Knowledge Q and A^a^	App or web	Knowledge explanation	Pomodoro study method
Tencent Cloud Presales Bot (Tencent Holdings Ltd)	Enterprise services	Web	Product consultation	AI^b^ functions in Tencent Meeting
Gaokao Xiaozhi (Zhangshang Gaokao)	Education	App or web	Academic counseling	University and major recommendation
Bank of China Online Bot (Bank of China)	Finance	Web	Banking consultation	Credit card selection
Ctrip Xiaoyou (Ctrip)	Travel	App or web	Booking assistance	Hotel room information
Huawei Xiaoyi (Huawei)	Consumer electronics	App	Technical support	Battery performance troubleshooting
Doubao (ByteDance)	Interview preparation	Web	Conversational assistance	Interview question coaching
Wenxin Yiyan (Baidu)	Education or assessment	Web	Question analysis	Exam question analysis
Kimi (Moonshot AI)	Health information	Web	Medical Q and A^a^	Hepatitis B test interpretation

a Q and A: question and answer.

b AI: artificial intelligence.

#### Sample and Data Collection

In July 2024, a pilot study involving 15 participants was conducted at a Beijing-based company, using convenience sampling. Participants completed the Chinese BUS-11 after performing designated tasks with selected chatbots. Completion times were recorded, and qualitative feedback regarding item clarity, ambiguity, and comprehension difficulties was collected through structured interviews. The pilot’s primary aims were to assess task feasibility, confirm average completion time, and identify any items requiring wording refinements. Pilot data were used only for refinement and were not included in the main validation dataset.

#### Outcomes Informing Quality Control

Based on the observed average completion time (~5 minutes 20 seconds), we set a priori a data-quality rule for the main study to exclude online questionnaires completed in less than 5 minutes, in addition to removing any incomplete submissions.

### Content Validity Validation

Between June and July 2024, 8 domain experts (HCI, informatics, chatbot engineering, and scale development; refer to Table 2) independently rated the relevance of each Chinese BUS-11 item to its intended dimension using a 4-point scale. Content validity was quantified to explicitly confirm item relevance and construct representativeness in the Chinese linguistic and cultural context following translation and cultural adaptation, even though no substantive changes were made to the original item content [19]. The item-level content validity index (I-CVI) was computed as the proportion of experts assigning ratings of 3 or 4, and the average scale-level content validity index (S-CVI) was calculated as the average of the I-CVIs. Criteria of I-CVI≥0.78 and S-CVI≥0.90 were used to indicate acceptable-to-excellent content validity. I-CVIs ranged from 0.875 to 1, with 4 items (items 3, 4, 6, and 11) achieving unanimous relevance (I-CVI=1). The average S-CVI was 0.920, indicating excellent content validity of the Chinese BUS-11. All content validity analyses were completed before the main psychometric validation study.

### Main Validation Study: Participants, Measures, Procedure, and Analysis

#### Participant Recruitment

Participants were recruited via social media platforms (eg, Red Note [Xiaohongshu Information Technology] and WeChat [Tencent Holdings Ltd]) according to predefined inclusion criteria: native Chinese speakers or individuals proficient in Chinese and aged 18 years or older. Recruitment followed an online convenience sampling approach; participation was voluntary. Each participant was randomly assigned to interact with 3 distinct chatbots (selected from the 10 systems), performing 1 predefined task per chatbot and completed the BUS-11 immediately after each interaction. Thus, each participant contributed 3 independent chatbot evaluations, yielding a repeated-measures data structure (evaluations nested within participants). For psychometric analyses (eg, reliability and exploratory factor analysis [EFA]), evaluations were analyzed at the evaluation level (N=438); design implications of repeated measures are discussed in the “Limitations” section.

#### Sample Size Estimation

Based on factor analysis requirements (5‐10 times the number of items, accounting for a 20% invalid rate), a minimum sample size of 69 participants was established. We targeted ≥70 participants and exceeded this target, ultimately recruiting 214 participants who provided 438 evaluations, thereby improving the robustness of reliability and factor-analytic estimates.

#### Participant Characteristics

We analyzed 438 valid chatbot evaluations submitted by 214 participants (69 males and 145 females), with each participant randomly assigned to evaluate 3 of 10 popular Chinese chatbot systems using the Chinese BUS-11. The sample skewed young, with 67.3% (144/214) aged 18‐24 years and 22.4% (48/214) aged 25‐30 years, and was highly educated, with 93% (199/214) holding a bachelor’s degree or above, and represented multiple industries, most prominently health care (52.8%, 113/214) and education or training (11.7%, 25/214). Most respondents had prior exposure to chatbots (79%, 169/214) and at least moderate familiarity (43%, 92/214) reported being familiar or very familiar, while 27.5% (59/214) reported being unfamiliar or very unfamiliar. These characteristics suggest a relatively tech-savvy cohort, which should be considered when interpreting generalizability. Detailed distributions are provided in Table 4.

**Table 4.** Characteristics of participants (N=214).

Characteristic and item	Participant, n (%)
Sex	
Male	69 (32.2)
Female	145 (67.8)
Age (years)	
18-24	144 (67.3)
25-30	48 (22.4)
31-40	14 (6.5)
41-50	5 (2.3)
51-60	3 (1.4)
Educational level	
Junior high school and below	2 (0.9)
High school	13 (6)
Bachelor’s degree	121 (56.5)
Master’s degree and above	78 (36.4)
Industry	
Manufacturing	9 (4.2)
Construction	4 (1.9)
Education or training	25 (11.7)
Internet	5 (2.3)
Health care	113 (52.8)
Computer	11 (5.1)
Finance	6 (2.8)
Scientific research	9 (4.2)
Government institutions	10 (4.7)
Professional consulting services	6 (2.8)
Culture and entertainment	8 (3.7)
Others	8 (3.7)
Familiarity with chatbots	
Very unfamiliar	26 (12.1)
Unfamiliar	33 (15.4)
Neutral	63 (29.4)
Familiar	61 (28.5)
Very familiar	31 (14.5)
Have you used a chatbot before?	
No	45 (21)
Yes	169 (79)
Frequency of using chatbots weekly	
Never (0)	53 (24.8)
Rarely (1-2)	69 (32.2)
Occasionally (3-4)	46 (21.5)
Often (5-6)	25 (11.7)
Every day (7)	21 (9.8)

#### Measures

Two primary instruments were used:

1. General demographic questionnaire: collected participant demographics and chatbot usage patterns.
2. Chinese BUS-11: a culturally adapted 11-item scale assessing chatbot usability across 5 dimensions. Because 2 BUS-11 dimensions (“Privacy and Security” and “Response Time”) are single-item constructs in the original instrument, they were retained in the survey but treated as single-item indicators in factor-analytic planning (refer to “Data Analysis” section).

#### Data Collection

Data were collected electronically via Tencent Questionnaire. Participants completed tasks on their own devices and then accessed the BUS-11 through a survey link. Strict criteria ensured data quality, and questionnaires that were not fully completed were directly discarded. Based on pilot timing, online questionnaires completed in less than 5 minutes were excluded to reduce insufficient-effort responses. In the pilot experiment, the research team measured the time taken to complete the questionnaire, with an average duration of 5 minutes and 20 seconds. The finalized main study dataset comprised 438 valid chatbot evaluations.

#### Data Analysis

Data were exported to Microsoft Excel, verified, and analyzed using IBM SPSS Statistics (version 26.0). Reliability was evaluated using Cronbach α coefficients for the overall scale and for each of its multi-item dimensions. Structural validity was assessed via EFA after sampling-adequacy checks (Kaiser-Meyer-Olkin [KMO] index and Bartlett test of sphericity). We used a principal components extraction with the Kaiser criterion (eigenvalues >1.0) and scree plot inspection to determine the number of factors and applied varimax rotation to aid interpretability. Standard criteria guided item retention: primary factor loadings of ≥0.40, absence of substantial cross-loadings (≥0.30), and conceptual interpretability of the resulting factor solution. Salient loadings were defined as those with values of ≥0.40. In line with the instrument’s structure, the EFA focused on the 9 items related to multi-item dimensions; the 2 single-item dimensions (“Privacy and Security” and “Response Time”) were not included in the EFA but were retained as single-item indicators in descriptive analyses. *P*<.05 was considered statistically significant for inferential tests (eg, Bartlett test of sphericity). We note that principal component analysis (PCA) is commonly used in early-stage exploratory work for data reduction and structure discovery, and future work may complement this with common-factor methods (eg, principal axis factoring) and confirmatory factor analysis (CFA) once larger samples are available.

### Ethical Considerations

This study was approved by the Ethics Committee of the School of Nursing, Peking Union Medical College (approval no PUMCSON-2023-10). All participants provided electronic informed consent before participation. Participation was voluntary, and participants could withdraw at any time without penalty. No personally identifying information was collected in the analytic dataset. Data were stored securely and analyzed in deidentified form.

## Results

### Scale Reliability and Validity

#### Structural Validity

**Figure 1.**
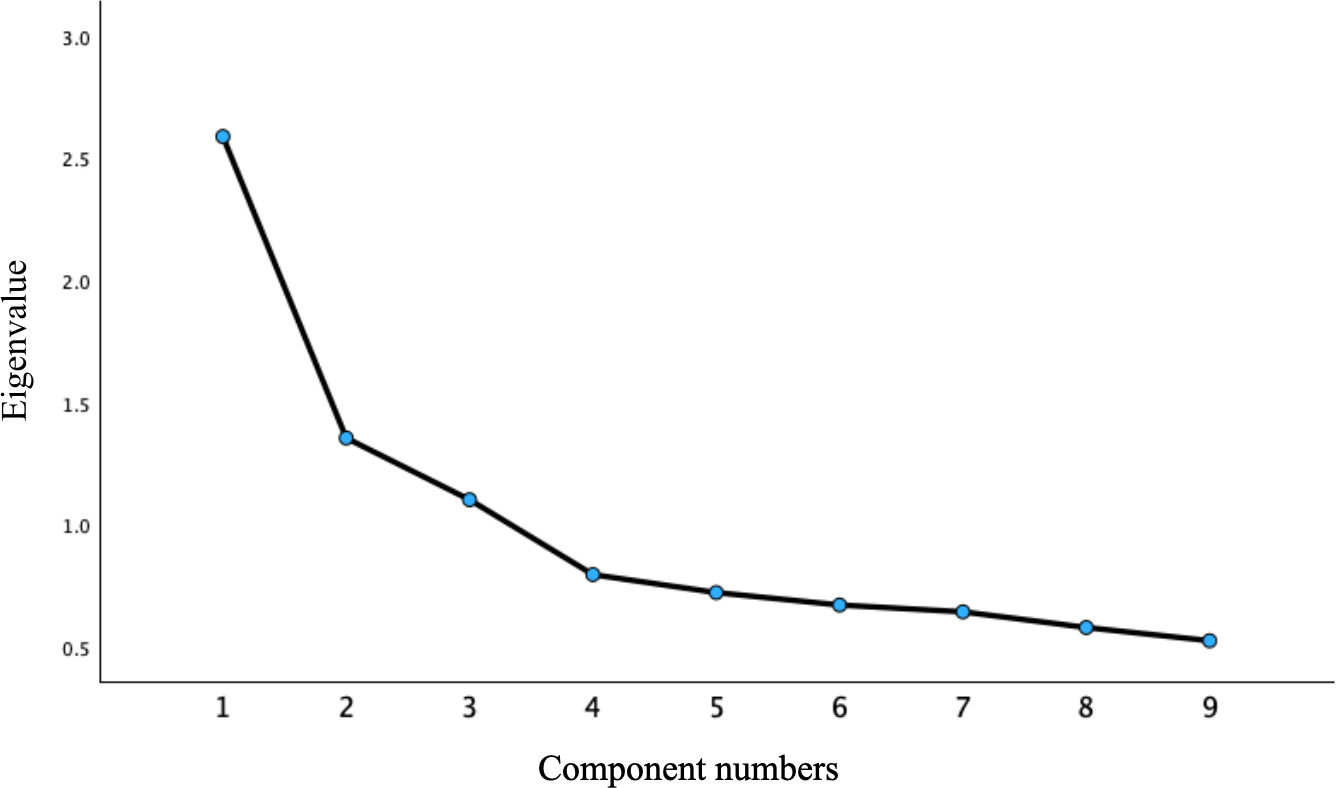
Scree plot of the factors.

Before factor extraction, data suitability was confirmed (KMO=0.743; Bartlett *χ*²_36_=628.185; *P*<.001) because 2 BUS-11 dimensions are single-item constructs (“Privacy and Security” and “Response Time”) and they were not included in the EFA. The analysis, therefore, used the remaining 9 items (refer to Figure 1 for the scree plot).

In this study, PCA and varimax orthogonal rotation were used to extract common factors. Nine items (items 10 and 11 were excluded from the EFA due to having only 1 item each) were analyzed and combined with the scree plot (Figure 1), and 3 common factors with eigenvalues greater than 1.0 were ultimately extracted, accounting for a cumulative variance contribution rate of 56.107%. The results of the EFA for this experiment are shown in Table 5.

**Table 5.** Exploratory factor analysis (EFA) loadings (3-factor solution) for the Chinese 11-item Chatbot Usability Scale (BUS-11).

Item	Factor		
	1	2	3
Access the chatbot’s functions			
Finding the entry to this chatbot is an easy task	0.840	—^a^	—
It is easy to find the various functions of this chatbot (eg, uploading a file, analyzing a file, changing chat avatar, and so on).	0.760	—	—
Quality of chatbot functions			
The communication process with this chatbot is smooth	—	0.757	—
This chatbot is able to maintain context coherence	—	0.759	—
The responses from this chatbot are easy to understand	—	0.716	—
Provide quality conversation and information			
I feel this chatbot understands my intentions and assists me in achieving the purpose of this chat	—	—	0.683
The information provided by this chatbot is appropriate, neither too much nor too little	—	—	0.627
This chatbot does not provide information relevant to my needs	—	—	0.629
I find the responses of this chatbot to be accurate and reliable	—	—	0.739

a Not available.

EFA with varimax rotation revealed a clear 3-factor structure for the Chinese version of BUS-11 (Table 5). The 3 factors explained 15.2%, 19.6%, and 21.3% of the total variance, respectively, accounting for a cumulative 56.1% of the variance. Factor 1 (Accessibility) consisted of 2 items reflecting the ease of locating and using chatbot functions, with strong loadings (0.840 and 0.760). Factor 2 (Interaction Process Quality) consisted of 3 items that described conversational smoothness, coherence, and comprehensibility, with loadings ranging from 0.716 to 0.759. Factor 3 (Information Quality) consisted of 4 items related to understanding user intent, the appropriateness of information, the absence of irrelevant content, and the reliability of responses, with loadings ranging from 0.627 to 0.739.

Only factors extracted by the eigenvalue >1 criterion are shown. Item domain labels reflect the original BUS-11 conceptual domains and are provided for reference.

#### Internal Consistency (Reliability)

The overall internal consistency of the Chinese BUS-11 was excellent (Cronbach α=0.92). By factor, reliability was acceptable to excellent, as indicated by Accessibility (Cronbach α=0.79), Interaction Process Quality (Cronbach *α*=0.91), and Information Quality (Cronbach α=0.93). Reliability was not computed for the single-item dimensions (“Privacy and Security” and “Response Time”). Item-level diagnostics (Table 6) showed corrected item-total correlations ranging from 0.453 to 0.819, indicating that all items were positively associated with the overall scale score. Cronbach α (if an item was deleted) ranged from 0.91 to 0.93, suggesting that removing any single item would not meaningfully improve internal consistency.

**Table 6.** Mean, SD, corrected item-total correlation, and Cronbach α (if an item is deleted) for the 11-item Chatbot Usability Scale (BUS-11).

Item	Values, mean (SD)	Corrected item-total correlation	Cronbach α (if an item is deleted)
1	3.79 (1.187)	0.539	0.93
2	3.57 (1.059)	0.616	0.92
3	3.77 (0.952)	0.819	0.91
4	3.77 (0.923)	0.785	0.91
5	3.79 (0.946)	0.816	0.91
6	3.66 (0.982)	0.817	0.91
7	3.57 (0.979)	0.785	0.91
8	3.53 (0.96)	0.771	0.91
9	3.55 (0.992)	0.798	0.91
10	2.80 (0.998)	0.453	0.93
11	4.08 (0.866)	0.469	0.93

### User Feedback on Item Clarity and Acceptability

Overall, participants reported that the Chinese BUS-11 was easy to understand and straightforward to complete. Most respondents indicated that the items were clear and adequately reflected their experiences with chatbot interactions. The average completion time was approximately 5 minutes, suggesting that the scale imposed a minimal response burden and was suitable for use in both research and applied settings without causing respondent fatigue.

While feedback was generally positive, one recurrent issue was identified. Participants provided optional open-ended comments on item clarity and completion experience. Comments were reviewed and summarized into recurring issues. Three themes emerged: (1) ambiguity in interpreting the privacy/security item, with some participants indicating uncertainty about what constitutes “risk disclosure” in chatbot interactions; (2) context-dependence of judgments, where respondents noted that perceived usability varied by task type and chatbot domain; and (3) overall clarity and low burden, with many respondents reporting that the items were easy to understand and could be completed quickly. These feedback themes informed our interpretation of the psychometric findings and priorities for future refinement.

### Sensitivity Analysis Across Sociodemographic Variables

To examine the robustness of the Chinese BUS-11 across user subgroups, we conducted sensitivity analyses by gender, age, and prior experience with chatbots (refer to Table 7). No significant differences in overall BUS-11 scores were observed between male and female participants (*P*=.12), nor across major age groups (*P*=.84), suggesting stable performance across these demographic characteristics. In contrast, participants with prior chatbot experience reported slightly higher BUS-11 scores than those without such experience (*P*=.03). This pattern indicates that the scale is sensitive to meaningful differences in user familiarity with chatbot interaction, while remaining robust across core sociodemographic variables.

**Table 7.** Sensitivity analysis of the 11-item Chatbot Usability Scale (BUS-11) scores across sociodemographic variables.

Variable and group	Value, mean (SD)	Statistical test	*P* value
Sex		*t* test	.12
Male	3.52 (0.80)		
Female	3.63 (0.69)		
Age (years)		One-way ANOVA	.84
18‐24	3.55 (0.87)		
25‐30	3.49 (0.95)		
31‐40	3.30 (0.67)		
41‐50	3.93 (0.67)		
Prior chatbot experience		*t* test	.03
Yes	3.64 (0.71)		
No	3.46 (0.78)		

## Discussion

### Rigorous Localization and Cultural Adaptation

This study localized the BUS-11 for the Chinese context, providing a standardized instrument for evaluating chatbot usability in China. Given BUS-11’s theory-driven and multidimensional design, its adaptation is particularly relevant for contemporary conversational systems. Following established cross-cultural adaptation practices, we applied a forward-backward translation workflow (inspired by the Brislin model) combined with iterative expert committee review to ensure semantic and conceptual equivalence [20]. The expert panel (HCI/human factors–oriented) focused on pragmatic clarity, idiomatic appropriateness, and cultural relevance, resulting in a Chinese BUS-11 that remained closely aligned with the original without substantive item revisions.

Notably, the absence of major item-level changes should not be interpreted as evidence that culture is irrelevant to chatbot usability. Instead, cultural effects may operate more subtly, shaping how users interpret and prioritize usability facets (eg, privacy expectations, responsiveness norms, and conversational politeness) rather than necessitating overt rewording. Such differences may be reflected in response patterns and the observed factorial organization, underscoring the importance of future work using multigroup approaches (eg, measurement invariance testing) and direct cross-cultural comparisons to examine whether BUS-11 functions equivalently across cultural groups and whether certain dimensions show differential salience in Chinese users. In addition, consistent documentation of translation decisions improves transparency and supports reproducibility for subsequent adaptation work and comparative HCI research [20]. Given the rapid expansion of chatbot deployment across service domains [21], a psychometrically grounded Chinese BUS-11 can facilitate rigorous UX benchmarking in China and enable more meaningful integration of Chinese user evidence into the broader HCI literature [22].

### Participant Sample and Representativeness

Our sample selection aimed for diversity in gender, age, education, and industry background, resulting in a broad cross-section of Chinese chatbot users. In practice, however, the achieved sample was skewed toward younger, highly educated adults. Most participants were aged 18‐30 years, and more than 90% held at least a bachelor’s degree. While this profile likely reflects the early adopters of new technologies [23] and those most engaged with contemporary chatbot applications, it also introduces some limitations on generalizability. Younger and tech-savvy users may have different usability perceptions than older or less technologically experienced individuals. Prior research in human factors has noted that older adults can face unique usability challenges and may interact with technology in distinct ways [24]. Our current sample underrepresents such groups, as well as users from rural areas or lower educational backgrounds, which means the usability impressions captured by the scale might not fully reflect the broader population of potential chatbot users. On the positive side, the concentration of experienced users in our sample could heighten sensitivity to subtle differences in chatbot interaction quality—these users are likely familiar with a variety of interfaces and may provide discerning feedback. The inclusion of participants from a range of industries (eg, education, health care, internet or information technology, and so on) is another strength, as it suggests the scale items were interpreted meaningfully across different usage contexts. Indeed, the BUS-11 was designed to be domain-agnostic, and our results tentatively support its applicability in multiple sectors. However, some fields (eg, finance and construction) were represented by relatively few respondents, so conclusions about those domains should be drawn cautiously. Likewise, because recruitment relied on convenience sampling (primarily in a few urban regions), there may be regional or cultural subgroup differences within China that our study did not capture. In summary, our sample provides a solid initial test of the scale among active chatbot users, but it is not fully representative of all user segments. Future research should broaden the sampling frame to include older adults, less frequent technology users, and a wider geographic distribution to ensure the Chinese BUS-11 is robustly validated for the general population. Broadening the participant base in this way would enhance the scale’s universality and address the western, educated, industrialized, rich, and democratic bias analog in the Chinese context, thus strengthening confidence that the tool works well for all target users, not just the young and educated subset.

### Reliability, Validity, and Key Psychometric Findings

#### Overview

We conducted a comprehensive psychometric evaluation of the Chinese BUS-11, including assessments of content validity, construct (factorial) validity, internal consistency reliability, and user feedback on item clarity and practical usability. Overall, the findings provide strong evidence that the localized scale retains the scientific integrity of the original instrument. Here, we discuss each set of findings in turn, relating them to established benchmarks and prior research, and interpret their implications for the scale’s use in HCI and human factors studies.

#### Content Validity

By consulting a panel of 8 experts to review the relevance of each item, we established that the content validity of the Chinese BUS-11 is high. The I-CVI for every item was very strong, with 4 items (Items 3, 4, 6, and 11) achieving an I-CVI of 1 (ie, unanimously deemed highly relevant by all experts). The remaining items also scored well above conventional acceptability thresholds [25]. Given that an I-CVI≥0.78 is often considered the minimum when 6 or more experts are involved, our results indicate that each item is considered clearly representative of the intended construct by domain specialists. The S-CVI was 0.920 (both by the averaging method and the universal agreement method), exceeding the common cutoff of 0.90 for excellent content coverage of a construct. These indices confirm that the BUS-11 (Chinese version) has comprehensive content coverage and the items collectively cover the important facets of chatbot usability without obvious omissions. This outcome was facilitated by the diverse expertise of our review panel, which included professionals in HCI, usability engineering, and systems engineering. Their varied perspectives helped ensure that the items were not only translated correctly but also conceptually appropriate for Chinese users. In practical terms, the high content validity suggests that Chinese BUS-11 can confidently be used to gauge chatbot usability aspects as intended—such as efficiency, clarity of answers, and user comfort—without missing key elements. The rigorous content validation process, in line with recommended scale development procedures, provides a solid foundation for the subsequent construct validation. It also offers reassurance to practitioners that the instrument has face validity in the local context (the items make sense to experts and presumably to end-users as well). We believe this strong content validity will help drive adoption of the scale in both research and industry evaluations, as stakeholders can trust that the instrument measures what it is supposed to measure (ie, salient user experience factors for chatbots).

#### Structural Validity (EFA)

To examine the underlying factor structure of the Chinese BUS-11, we carried out an EFA on the survey responses. Preliminary checks confirmed that our data were suitable for factor analysis: the KMO measure of sampling adequacy was 0.743, which is above the usual threshold of 0.60 and can be considered “middling” to “good” [26], and the Bartlett test of sphericity was highly significant, indicating sufficient interitem correlations for extracting latent factors. Using principal component extraction with varimax rotation, we identified a 3-factor solution that best fit the data. These 3 extracted common factors had eigenvalues greater than 1 and together accounted for about 56.1% of the total variance in users’ responses. A cumulative variance above approximately 50% is acceptable in behavioral research, given the complexity of HCI measures, so 56% indicates that the scale captures a substantial portion of the usability perception variance in our sample. Each factor showed a clear thematic grouping of items, reflecting distinct dimensions of the chatbot user experience. Notably, 2 specific usability aspects (ie, “Privacy and Security” and “Response Time”) were each originally measured by a single item in the BUS-11. As single-item facets, they could not load onto multi-item factors in the EFA and were thus analyzed separately (we examined their scores independently rather than including them in the factor structure). The remaining nine items clustered into three coherent factors: (1) Accessibility (eg, ease of accessing and using the chatbot, such as simple interface and low effort to start the interaction), (2) Interaction Quality (covering the smoothness, coherence, and understandability of the conversational exchange), and (3) Information Quality (assessing the relevance, clarity, and correctness of the chatbot’s responses, without unnecessary redundancy). This structure is broadly consistent with the original conceptual framework of the BUS scale, which encompassed multiple dimensions of chatbot usability [10], but our findings suggest a somewhat more consolidated factor model in the Chinese context.

In the original development of the BUS, a 5-factor model (BUS-15) was proposed, which included separate dimensions for privacy and response speed alongside factors analogous to those we found [10]. Our EFA results imply that Chinese users may not distinguish as many separate categories, instead perceiving a more integrated set of usability factors. Attributes related to the conversational process and functional outcome appear to intertwine; users who find the interaction process smooth also tend to perceive the information provided as high quality, suggesting an underlying general perception of “interaction effectiveness.” Interestingly, a recent re-examination of BUS-11 in a large multichatbot dataset also identified a simpler factor structure than initially theorized [27]. Our findings align with that perspective, reinforcing the idea that a parsimonious model (fewer and broader factors) can sufficiently capture chatbot usability evaluations. It is worth noting that we chose to retain the “Privacy and Security” and “Response Time” items as standalone indicators in the instrument, due to their importance in usability (users do care about privacy and speed) and their presence in the original scale. However, from a psychometric standpoint, single-item factors are suboptimal because they do not allow for internal consistency reliability estimation and cannot capture the breadth of a construct. Best practices in scale development typically recommend having at least 2‐3 items per factor to achieve adequate reliability and construct representation [28]. Therefore, one implication is that future refinement of the Chinese BUS might involve expanding these facets—adding a couple of items to better gauge the privacy/security aspect of chatbot use (eg, covering data protection, user consent, and so on) and the responsiveness aspect (eg, not just speed but also perceived responsiveness or promptness). For now, including those single items ensures that our localized scale does not lose any content relative to BUS-11, and they provide useful standalone measures (eg, designers might specifically want to see ratings of privacy transparency). In summary, the EFA supports the structural validity of the Chinese BUS-11 by confirming that its items coherently measure multiple distinct dimensions of usability. The identified 3-factor structure captures the major themes of chatbot usability experience in our sample. This structure provides an empirical basis for scoring or interpreting the scale (eg, computing subscale scores for Accessibility, Interaction Quality, and Information Quality) and sets the stage for future confirmatory testing to verify whether this structure generalizes to other samples or holds under more stringent statistical criteria.

#### User Feedback on Item Clarity

The observed variability in interpreting the privacy/security item suggests that privacy-related usability judgments may be more sensitive to users’ privacy literacy and to how risk information is surfaced in routine interactions, consistent with prior privacy research indicating limited user engagement with privacy policies and disclosures [29,30]. From a measurement perspective, this supports retaining privacy/security as a relevant usability facet while motivating future refinement (eg, adding additional items or more concrete wording) and the use of cognitive interviewing to improve interpretability across user subgroups [31]. In addition, the brief completion time (≈3‐4 minutes) suggests that the Chinese BUS-11 imposes low respondent burden, comparable to widely used usability instruments and suitable for rapid assessment in applied settings [32,33].

### Limitations

While this study yielded encouraging results for the Chinese adaptation of BUS-11, several limitations must be acknowledged to contextualize the findings and guide future work. First, the sample, as discussed, was not fully representative of all user demographics. The geographic distribution of participants was relatively narrow (with many users from a few major cities), and certain age groups (especially adults aged older than 50 years) and occupations were underrepresented. The current sample underrepresents such groups, as well as users from rural areas or lower educational backgrounds, which means the usability impressions captured by the scale might not fully reflect the broader population of potential chatbot users. Indeed, the BUS-11 was designed to be domain-agnostic, and the results tentatively support its applicability in multiple sectors. However, some fields (eg, finance and construction) were represented by relatively few respondents, so conclusions about those domains should be drawn cautiously. Likewise, because recruitment relied on convenience and snowball sampling (primarily in a few urban regions), there may be regional or cultural subgroup differences within China that this study did not capture. This means the current validation is most directly applicable to younger, educated Chinese users in urban settings. Caution should be used in generalizing the psychometric results to, say, older adults or rural users, who might interact with chatbots differently or have different usability concerns. Expanding the sample in future studies will be important to verify that the scale maintains its reliability and validity across a broader swath of the Chinese population. Additionally, larger and more heterogeneous samples would allow for analyses of measurement invariance—checking whether the scale functions equivalently across subgroups such as older vs younger users, or high-frequency vs low-frequency chatbot users [34]. In addition to representativeness concerns, the overall sample size should be considered a methodological limitation. Although the study included 438 chatbot evaluations, these ratings were provided by 214 participants, which may be modest for robust factor-analytic procedures. According to the International Test Commission Guidelines for Translating and Adapting Tests [35], stable and generalizable factor solutions are typically supported by larger participant samples. The relatively limited number of participants in this study may therefore constrain the stability and generalizability of the extracted factor structure and other psychometric estimates. Future studies with larger and more diverse samples are needed to further confirm the robustness of the Chinese BUS-11’s factor structure and measurement properties.

Second, certain BUS-11 dimensions in the current form rely on a single item (notably the “Privacy and Security” and “Response Time” facets). Single-item measures, while convenient, provide limited psychometric information. They can neither capture the full breadth of a construct nor estimate internal consistency for that construct. The strong performance of the overall scale notwithstanding, the precision and reliability of those facets could be improved. Future research should consider enriching these dimensions by developing and testing additional items. For example, the privacy facet might be expanded with items addressing whether the chatbot clearly asks permission for data usage or whether it provides settings for privacy control. The response time facet might include an item on whether the chatbot’s speed meets user expectations or if delays ever cause frustration. Of course, any new items would themselves need to undergo translation and validation. The trade-off is that adding items could slightly increase completion time, but as long as the total remains reasonable (eg, <2 minutes increase), it would likely be worth it for a more robust scale. In short, the current findings highlight the need to bolster those single-item constructs for a more rigorous measurement model [28].

Third, this study primarily evaluated the scale in general usage scenarios (common chatbot systems for generic tasks) and did not deeply examine how the scale performs in specific industry contexts or use-case scenarios. It remains an open question whether users in specialized domains—such as health care, education, finance, or customer service—interpret and prioritize the BUS-11 items similarly. Different contexts may put extra emphasis on certain usability aspects; for instance, in health care chatbot applications, issues such as empathy, trust, and accuracy of information might be even more critical [36], whereas in e-commerce chatbots, speed and transactional clarity could dominate user satisfaction. The inability to test such variations is a limitation. It is possible that the BUS-11 might require minor tweaks or weighting adjustments to be optimally effective in certain domains, or it may prove to be robust as is—we simply do not have the data yet to say. Future validations should include a variety of chatbot types and domains to ensure the scale’s content validity and structure hold in those settings, or to highlight any domain-specific gaps that could be addressed by additional context-specific items.

Additionally, this study did not use external, established usability instruments to assess convergent validity. Although the BUS-11 is conceptually grounded in usability theory, the absence of a comparator scale—such as the System Usability Scale or other widely used usability measures—limits the ability to empirically examine whether the Chinese BUS-11 correlates appropriately with validated measures of related constructs. As a result, the current validity evidence is primarily restricted to internal structure and reliability and does not allow for a direct assessment of alignment with existing usability benchmarks. Future studies should consider incorporating one or more established usability scales to evaluate convergent (and potentially discriminant) validity, thereby strengthening the overall validity framework and situating the Chinese BUS-11 more clearly within the broader landscape of usability measurement tools. Moreover, test-retest reliability was not assessed due to the cross-sectional design. Therefore, the temporal stability of the Chinese BUS-11 could not be directly evaluated. Future studies should administer the scale to the same users across time points to examine score stability when the usability of a chatbot system remains unchanged.

Finally, the psychometric analysis relied on EFA due to the novelty of applying the BUS-11 in Chinese. We have not yet conducted a CFA on a separate sample, which would be the next step to confirm whether the 3-factor model (plus the 2 single-item factors) holds under stricter modeling conditions. A CFA would allow us to test the hypothesized factor structure (possibly including correlated factors or a higher-order factor of overall usability) and to adjust for measurement errors [37]. It would also enable a formal comparison of alternative models—for example, whether a structure with 5 distinct factors (if privacy and speed were expanded) fits significantly better than a 3-factor structure, or whether a single-factor model (treating usability as unidimensional) is decisively inferior to a multifactor model. Additionally, with a large enough sample, multigroup CFA could be used to test measurement invariance across key groups (eg, male vs female, younger vs older, and different regions), ensuring the scale operates equivalently [34]. Because the current sample size was modest for CFA and was collected as a single group, we reserve these confirmatory analyses for future work. In summary, the use of EFA was appropriate for initial validation; however, further confirmation via CFA is needed to solidify the factor structure and verify that the Chinese BUS-11 meets the standards of psychometric validity expected for a widely applicable research instrument.

The exploratory factor analytic results should be interpreted considering the factor extraction approach used in this study. We note that PCA is commonly applied in early-stage exploratory research for data reduction and initial structure discovery; however, PCA does not explicitly model latent constructs. As such, while appropriate for an initial examination of the scale structure, this approach provides only preliminary evidence regarding the underlying factor model. Future research with larger samples may complement these findings by applying common-factor methods (eg, principal axis factoring) and conducting CFA to more rigorously test the latent structure of the Chinese BUS-11.

### Conclusion

This study delivers a validated Chinese adaptation of BUS-11 with excellent internal consistency, strong expert-rated content validity, and a clear 3-factor structure for multi-item facets (Accessibility, Interaction Process Quality, and Information Quality), while preserving the privacy and speed facets conceptually. The instrument fills a measurement gap for chatbot UX evaluation in China and provides researchers and practitioners with a concise, psychometrically sound tool suitable for laboratory and field contexts alike. Future work should establish temporal stability, convergent validity, and confirmatory structure and expand coverage to underrepresented user groups and domain-specific scenarios to further strengthen its utility and generalizability.

## Supplementary material

10.2196/84971 Multimedia Appendix 1 Standardized user tasks applied across 10 popular chatbot systems in China to ensure consistency and ecological validity in usability evaluations with the Chinese 11-item Chatbot Usability Scale (BUS-11).

## Abbreviations

BUS-11: 11-item Chatbot Usability Scale
CFA: confirmatory factor analysis
CUQ: Chatbot Usability Questionnaire
EFA: exploratory factor analysis
HCI: human-computer interaction
I-CVI: item-level content validity index
KMO: Kaiser-Meyer-Olkin
PCA: principal component analysis
S-CVI: scale-level content validity index
UX: user experience
