# Supplementary material for: Chatbot Usability Scale in Chinese Users: Cross-Cultural Adaptation and Validation Study
Source: JMIR Hum Factors. 2026 Apr 6;13:e84971. doi: 10.2196/84971 (PMC13052771; doi:10.2196/84971)
Supplement: Multimedia Appendix 1 [file humanfactors-v13-e84971-s001.docx]

**1. Taobao – Understanding 88VIP Membership Benefits**

Background: A friend has recommended that you sign up for Taobao’s 88VIP membership program. You want to understand what specific benefits and privileges this 88VIP membership offers.

Goal: To use Taobao’s chatbot to obtain detailed information about the 88VIP membership benefits.

Action Steps:

• Navigate to the Taobao website and log in to your account (if you are not already logged in).

• Find and initiate a chat with Taobao’s customer service chatbot, “Smart Customer Service XiaoMi.”

• Ask the chatbot to provide details about the benefits of the 88VIP membership program.

• Read and note the membership benefits information provided by the chatbot.

• After completing the conversation, evaluate the chatbot’s usability using the given usability scale.

**2. Zhangshang Gaokao – Choosing a Suitable University for a Nursing Major**

Background: You have recently taken China’s National College Entrance Examination (Gaokao) and received a score of 596. You are from Beijing and your elective subjects were Physics, Chemistry, and Biology. Now, as you fill out your college application, you wish to pursue a major in Nursing.

Goal: To identify five universities that offer a Nursing program and are the best fit for your score and academic profile, using the “Gaokao Xiaozhi” chatbot on the Zhangshang Gaokao admissions platform.

Action Steps:

• Open the Zhangshang Gaokao college admissions guidance platform (via its website or mobile app).

• Access the platform’s chatbot, “Gaokao Xiaozhi.”

• Provide your Gaokao score (596), location (Beijing), and intended major (Nursing) to the chatbot.

• Ask the chatbot to recommend five universities that would be most suitable for you given your score and preferences.

• Review the list of recommended universities (and any advice) provided by the chatbot.

• After completing the task, evaluate the chatbot’s usability using the provided scale.

**3. Tencent Meeting – Inquiring About AI Features**

Background: You are interested in the new AI-powered features of Tencent Meeting (a video conferencing platform) and want to learn what these features do and how to use them.

Goal: To understand the purpose of Tencent Meeting’s AI features and how to use them, by interacting with Tencent’s official customer support chatbot.

Action Steps:

• Navigate to the Tencent Meeting service page on the Tencent Cloud website.

• Log in using your WeChat account when prompted (to access the support system).

• Locate and start a conversation with the “Pre-sales Consultation” chatbot (Tencent’s customer support virtual assistant for Tencent Meeting).

• Ask the chatbot about the functions of Tencent Meeting’s AI features and how to use these features within the application.

• Read and note the explanations and usage instructions provided by the chatbot.

• Upon concluding the chat, evaluate the chatbot’s usability with the given usability scale.

**4. Zhihu – Learning about the Pomodoro Technique**

Background: You are a second-year university student who will take a final exam in Marxist Philosophy next month. Seeking to improve your study efficiency, you have heard from a classmate about the Pomodoro Technique for time management.

Goal: To understand what the Pomodoro Technique is and how to apply it to your study plan for the Marxist Philosophy course, by consulting Zhihu’s AI-driven Q&A service (the “Zhihu ZhiDa” chatbot).

Action Steps:

• Visit Zhihu, a Chinese knowledge-sharing platform, and log in to your account.

• Access the “Zhihu ZhiDa” feature (an AI-based question-answering assistant on Zhihu’s platform).

• Ask the chatbot to explain the Pomodoro Technique and how it can be implemented in your revision plan for the Marxist Philosophy exam.

• Review the detailed explanation and study strategy tips provided by the chatbot.

• After using the service, evaluate the chatbot’s usability using the provided scale.

**5. Baidu Wenxin Yiyan – Analyzing Gaokao Exam Content**

Background: You are a high school student in Shanghai preparing for the 2025 National College Entrance Examination (Gaokao). Past Gaokao exam answers are not officially published, so you seek alternative study resources. You decide to use Baidu’s Wenxin Yiyan chatbot to help review prior exam material.

Goal: To retrieve and analyze the reading comprehension section of the 2023 Shanghai Gaokao Chinese exam using the Wenxin Yiyan AI chatbot.

Action Steps:

• Go to Baidu’s AI service portal and access the Wenxin Yiyan chatbot (Baidu’s large-language-model-based assistant).

• In the chatbot interface, request the reading comprehension section of the 2023 Shanghai Gaokao Chinese exam and ask for an analysis of that section.

• Wait for the chatbot to retrieve the relevant exam text and provide an explanation or analysis of the reading comprehension questions and answers.

• Read the analysis given by Wenxin Yiyan, noting any insights or details it provides about the exam section.

• After concluding this session, evaluate the chatbot’s usability using the given scale.

**6. Doubao – Getting Advice on an Interview Question**

Background: You are preparing for an upcoming online job interview and feel anxious. One practice question you have encountered is: “In a meeting with five managers, only four bottles of water were provided. What would you do?” This question is meant to test your problem-solving and interpersonal skills under pressure.

Goal: To develop a strong, logical answer to the interview question—and identify key points that will make you stand out as a candidate—by consulting the Doubao chatbot.

Action Steps:

• Open the Doubao chatbot platform (an online AI assistant for query-based problem solving).

• Present the interview scenario to the chatbot: explain that there are five managers in a meeting but only four bottles of water available, and ask how to handle this situation professionally.

• Request the chatbot to outline a logical approach for addressing the problem and to highlight important elements that would make your response impressive to interviewers.

• Review the solution and the highlighted key points provided by the chatbot for crafting your answer.

• After the interaction, evaluate the chatbot’s usability using the provided scale.

**7. Kimi – Clarifying Medical Test Results**

Background: You recently underwent a medical check-up, and your hepatitis B lab results came back with confusing findings: one hepatitis B antibody test is positive, while the hepatitis B surface antibody and core antibody tests are negative. You are uncertain whether this means you have (or had) a hepatitis B infection and whether you should get vaccinated.

Goal: To interpret your hepatitis B test results and determine if you need a hepatitis B vaccination, by consulting the Kimi online health chatbot.

Action Steps:

• Access the Kimi medical consultation chatbot platform.

• Provide the chatbot with the key details of your test results (e.g., a positive hepatitis B antibody test, with negative surface and core antibody tests).

• Ask the chatbot whether these results indicate a hepatitis B infection (current or past) and inquire if you should receive a hepatitis B vaccine.

• Read the explanation and medical advice given by the chatbot regarding your condition and recommended next steps (such as vaccination).

• After completing the consultation, evaluate the chatbot’s usability using the provided scale.

**8. Huawei – Resolving a Smartphone Battery Issue**

Background: You use a Huawei Mate60 Pro smartphone and have noticed a decline in its battery performance, especially during charging. The phone charges more slowly than before, and the battery seems to drain quickly. You want to address this issue.

Goal: To find troubleshooting advice or a solution for the battery performance problem by interacting with Huawei’s intelligent support chatbot (“Smart Customer Service Xiaoyi”).

Action Steps:

• Go to Huawei’s official support website and log into your Huawei account (register a new account if you do not have one).

• Locate and launch the “Smart Customer Service Xiaoyi” chatbot on the support site.

• Describe the battery issue to the chatbot, including details like your phone model (Mate60 Pro) and the observed symptoms (slow charging and reduced battery performance).

• Ask the chatbot for guidance on how to improve the battery performance or fix the charging issue.

• Review the troubleshooting advice or steps provided by the chatbot to address the problem.

• Once the session is finished, evaluate the chatbot’s usability using the provided scale.

**9. Bank of China – Finding a Suitable Credit Card for Travel**

Background: You are a resident of China planning a two-week trip to Thailand during the summer of 2025. To prepare, you want a credit card that can be used conveniently in Thailand, and you need to know which card to get and how to apply for it.

Goal: To determine which type of credit card offered by Bank of China can be used in Thailand and to learn how to apply for it, by using the bank’s “Online Customer Service” chatbot.

Action Steps:

• Visit the official Bank of China website.

• Find and initiate a chat with the “Online Customer Service” chatbot on the site.

• Ask the chatbot which credit card options are suitable for use in Thailand during a short trip.

• Inquire about the features of the recommended credit card (such as international usage fees or benefits) and ask for instructions on how to apply for that credit card.

• Note the information and guidance provided by the chatbot regarding the credit card selection and application process.

• After obtaining the information, evaluate the chatbot’s usability using the provided scale.

**10. Ctrip – Checking Hotel Room Amenities via Chatbot**

Background: You are traveling from Shijiazhuang (Hebei Province) to Beijing for a summer vacation. You have booked a Deluxe Double King Room at the Beijing Hotel through the Ctrip platform, and you want to confirm the details of this room. In particular, you need to know whether the room has a refrigerator.

Goal: To verify if the Deluxe Double King Room at Beijing Hotel (booked via Ctrip) includes a refrigerator, by querying Ctrip’s “Xiao You” customer service chatbot.

Action Steps:

• Go to the Ctrip website (or open the Ctrip app) and log in to your account.

• Navigate to the customer support or help section and launch the “Xiao You” virtual assistant (Ctrip’s chatbot).

• Provide the chatbot with your booking details (Beijing Hotel, Deluxe Double King Room) and ask whether this room type includes a refrigerator among its amenities.

• Review the information given by the chatbot about the room’s amenities, noting specifically if a refrigerator is listed.

• Upon resolving your query, evaluate the chatbot’s usability using the provided scale.
